# Tensile Properties of Ex-Situ Ti-TiC Metal Matrix Composites Manufactured by Laser Powder Bed Fusion

**DOI:** 10.3390/ma17225613

**Published:** 2024-11-17

**Authors:** Gaëtan Bernard, Vaclav Pejchal, Olha Sereda, Roland E. Logé

**Affiliations:** 1 Additive Manufacturing and Component Reliability Group, CSEM SA, Rue Jaquet-Droz 1, CH-2002 Neuchâtel, Switzerland; vaclav.pejchal@csem.ch; 2 Thermomechanical Metallurgy Laboratory, PX Group Chair, Ecole Polytechnique Fédérale de Lausanne (EPFL), CH-2002 Neuchâtel, Switzerland; roland.loge@epfl.ch; 3 Etat de Neuchâtel, Rue de la Collégiale, CH-2002 Neuchâtel, Switzerland

**Keywords:** metal matrix composite, titanium alloy, titanium carbide, additive manufacturing, mechanical properties, microstructural properties

## Abstract

Titanium-based Metal Matrix Composites (MMCs) manufactured by additive manufacturing offer tremendous lightweighting opportunities. However, processing the high reinforcement contents needed to substantially improve elastic modulus while conserving significant ductility remains a challenge. Ti-TiC MMCs fabricated in this study reported fracture strains in tension up to 1.7% for a Young’s modulus of 149 GPa. This fracture strain is 30% higher than the previously reported values for Ti-based MMCs produced by Laser Powder Bed Fusion (LPBF) displaying similar Young’s moduli. The heat treatment used after the LPBF process leads to the doubling of the fracture strain thanks to the conversion of TiC_x_ dendrites into equiaxed TiC_x_ grains. The as-built microstructure shows both un-dissolved TiC particles and sub-stoichiometric TiC dendrites resulting from the partial dissolution of TiC particles. The reduction of the C/Ti ratio in TiC during the process results in an increase in the reinforcement content, from a nominal 12 vol% to an effective 21.5 vol%. The variation of the TiC lattice constant with its stoichiometry is measured, and an empirical expression is proposed for its effect on TiC’s Young’s modulus. The lower TiC powder size distribution displayed higher mechanical properties thanks to a reduced number of intrinsic flaws.

## 1. Introduction

Since its introduction, Laser Powder Bed Fusion (LPBF) has played a significant role in refining Titanium alloys’ exceptional properties in terms of ductility, stability at high temperatures, specific strength and resistance to corrosion. Standing out within the metal Additive Manufacturing (AM) landscape, the LPBF method offers a greater degree of flexibility in terms of shapes, as well as the potential to reduce the total number of components of a part compared to traditional metallurgical processes. Moreover, this technology is particularly suited for medical and aerospace applications, as the higher cooling rates occurring in LPBF compared to conventional technologies limit grain growth, i.e., between $10^{4}\;K/s$ and $10^{6}\;K/s$ for LPBF, while conventional methods such as casting and powder metallurgy rarely surpass $10^{2}\;K/s$ [1]. The strongly refined microstructure induced generally presents increased strength, wear resistance and toughness.

However, further enhancement of mechanical properties can be obtained by reinforcing Ti alloys with a second component, usually ceramic, and producing a Metal Matrix Composite (MMC). These materials are promising candidates for increasing and controlling specific stiffness. Titanium MMC has been produced by conventional methods and has demonstrated remarkable mechanical properties [2]. As an example, Self-propagating High-temperature Synthesis (SHS) and Spark Plasma Sintering (SPS) were used by Lagos et al. to produce Ti64-TiC MMC with a Young’s modulus improvement of 15%, while keeping the fracture strain above 3% [3]. However, due to the hardness difference between the matrix and the reinforcement, MMCs are usually difficult to machine and are confined to simple shapes. In addition, the hard phase causes a high tool wear rate, leading to high machining costs. Therefore, AM triggers a radical change for MMC manufacturing due to its ability to produce neat net shapes, as demonstrated by the number of publications in the field in the last 15 years.

Several reviews covering the recent advances in the use of LPBF to fabricate Ti-based MMCs have been published in recent years [4,5,6] and give insight into the researcher’s focus points. First, the main reinforcements for Ti-based MMCs are TiC and TiB and can be introduced directly as TiC and TiB_2_ or by using C and B precursors, which will react with Ti to form carbides and borides. These reinforcements are favoured thanks to their coefficient of thermal expansion being relatively close to that of Ti, which reduces thermal residual stresses, reducing the solidification cracking risk. TiB is reported to have a higher strengthening effect than TiC at an equivalent reinforcement content but to induce a larger ductility decrease [5]. Second, a majority of the studies focus on strength and wear resistance enhancements, which require lower reinforcement contents than elastic modulus enhancement. Third, producing a significantly strengthened MMC displaying significant elongation is still challenging. This is reflected by the minority of studies reporting ductility measurements such as elongation at break values. For example, Xi et al. worked on Ti reinforced by 30 vol% of TiC, TiN, a mix of both, as well as graphene [7]. They could produce parts with each type of powder mix but could not produce Ti-TiC and Ti-TiN samples without severe cracking. Moreover, they also studied the hardness of the achieved MMCs and showed that TiC produces the highest strengthening effect. Radhakrishnan et al. alternatively demonstrated the possibility of producing crack-free Ti-TiC coatings with up to 20 vol% reinforcement content by LMD [8]. While the samples displayed a brittle failure and the elastic modulus was not characterized, it opens the possibility of reaching reinforcement content of the order 20 vol% in bulk components. Using nanoindentation tests, Gu et al. demonstrated a very promising stiffness increase in LPBF-produced Ti reinforced by 15 wt% TiC nanoparticles, which displayed a nano-hardness of 90.9 GPa with a local reduced modulus of 256 GPa [9,10]. While there is no indication of the ductility of the produced MMC, it does display the potential of such reinforcement content. Yan et al. reinforced Ti6Al4V with graphene by LPBF and, despite the low 0.5 wt% of reinforcement content [11], could achieve a strong strengthening effect with an Ultimate Tensile Strength (UTS) of 1526 MPa and Young’s modulus of 145 GPa while displaying a fracture strain of 1.3%. Additionally, using the identical powder mix and reinforcement content, the same study compared these results with parts produced with SPS, for which the strengthening effect was much lower, as demonstrated by the latter displaying a UTS of 877 MPa, Young’s modulus of 115 GPa and fracture strain increase of 3.9%. They assumed that the discrepancy was due to graphene reacting at elevated temperatures with Ti to form TiC, and proposed that the short process time scale in LPBF prohibited the reaction from occurring. While it is relatively low, the reported 1.3% fracture strain is, to the authors’ knowledge, the highest reported for Ti-based MMCs produced by AM technologies that display significant stiffness improvement [11]. Ti-based MMCs produced by conventional methods have been reported as early as the 2000s to achieve elongation of 2.5 to 3% for such elastic moduli, which demonstrates the need for further research on the subject [2]. This study focuses on reducing the gap between the fracture strains achieved by LPBF and conventional methods for stiffness-driven Ti-based MMCs.

TiC in Ti-TiC MMCs is known for its deviation from the equilibrium phase diagram in terms of the C/Ti ratio. Andrieux et al. and Roger et al. studied the evolution of this ratio in Ti-TiC MMCs during heat treatment and observed an experimentally stable C/Ti ratio of 0.57 in the TiC phase [12,13]. Lin et al. demonstrated an inverse relation between the cooling rate and the obtained C/Ti ratio [14]. The variation of the C/Ti ratio affects the effective reinforcement content through the distribution of the C atoms over a larger number of TiC molecules but also a variation of the lattice constant. Moreover, mechanical properties are also impacted by this variation. Kurlov and Gusev compiled the available data and proposed empirical relations for the lattice constant, shear modulus and Poisson ratio as a function of the C/Ti ratio [15]. Hence, it is recommended to study the evolution of the reinforcement from the powder stage (nominal volume fraction) to the finished MMC (effective volume fraction), which is often neglected in mechanical-properties-related studies. Referring only to the nominal content of reinforcement may significantly underestimate the volume fraction depending on the achieved stoichiometry.

This study aims at improving the fracture strain in tension of a Ti-TiC MMC displaying a significantly enhanced Young’s modulus. It uses a 3-step process consisting of mechanical blending, LPBF process and heat treatment, and it investigates several size distributions of TiC powders to determine the optimal one for stiffness-driven Ti-TiC MMCs. The control of the reinforcement geometry through the addition of a heat treatment and its effect on ductility in investigated. In order to ensure the practicality and scalability of the process, we focus on readily available and easily producible forms of TiC: comminuted TiC powders. For the same reason, the mechanical properties are characterized by tensile testing on near-net-shape ASTM E8M compliant tensile samples [16].

## 2. Materials and Methods

**Figure 1:**
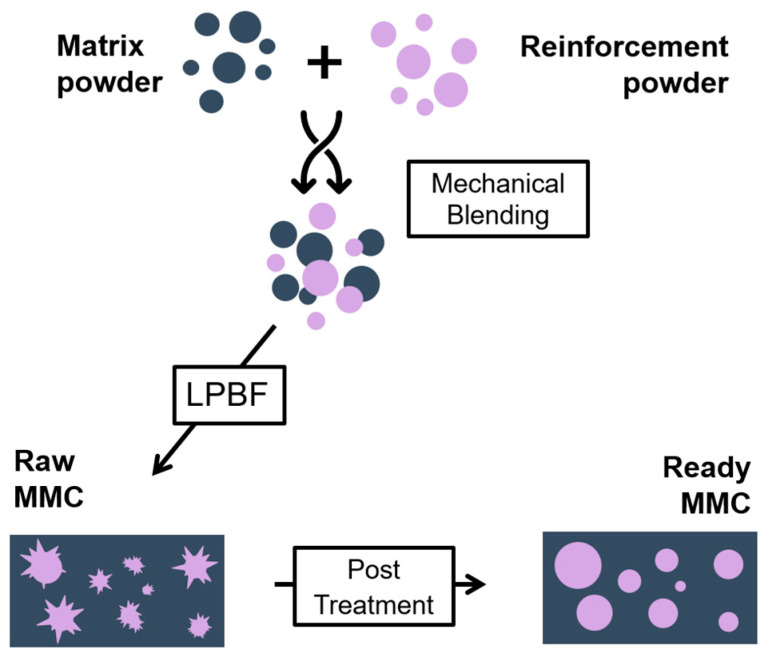
MMC Manufacturing process.

The full metal matrix composite manufacturing process is explained in detail in the next sections and is summarized in Figure 1. It consists of a powder preparation step in which a premix is prepared mechanically, followed by the LPBF process used to consolidate the MMC. The dendritic TiC microstructure is then globularised through heat treatment, leading to a stabilized microstructure.

### 2.1. Powder Preparation

**Figure 2:**
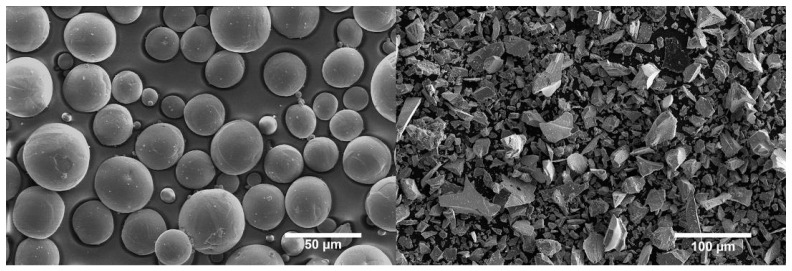
SEM micrographs presenting (**a**) Plasma atomised spherical Titanium powder, (**b**) Comminuted Titanium Carbide Powder.

**Figure A1:**
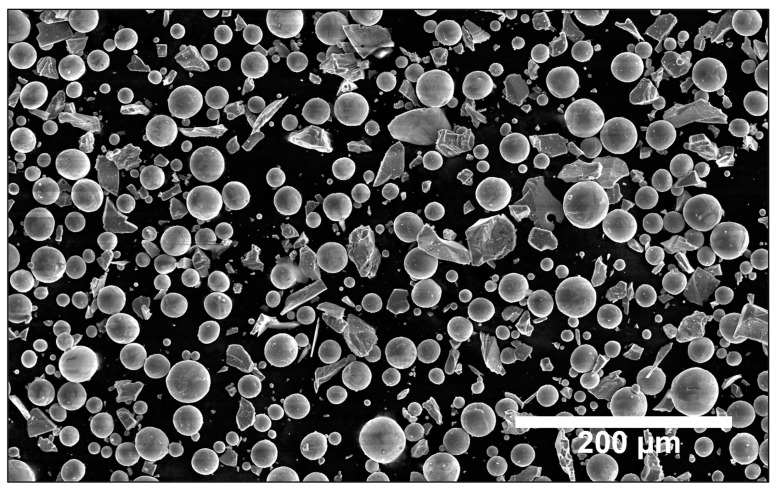
SEM micrograph of typical Ti-TiC powder mix.

The powder mixtures include plasma atomized spherical commercially pure Titanium (Cp-Ti grade 2, 5–45 µm, Figure 2a) from AP&C and comminuted 99% purity Titanium Carbide powder (TiC, 5–45 µm, Figure 2b) from Höganäs GmbH (Hoganas, Sweden). The powders’ chemical compositions are available in the Appendix A, Table A1. The comminuted TiC powder is sieved in two different ways, producing two size distributions: one with an upper threshold of 45 µm, and the other with a threshold of 23 µm. The first sieving removes all agglomerates from transport and leads to the full 5–45 µm distribution. The second one effectively removes all particles larger than 23 µm. Two powder blends containing 87 wt% cp-Ti and 13 wt% of TiC are prepared by means of a 3D shaker mixer from the WAB Group (Turbula T2F, Muttenz, Switzerland) for at least 3 h. This blending method uses a motion in three dimensions of the powder container to gently mix the two powders together and avoid particle damage. The powder blends using comminuted TiC sieved below 45 µm and below 23 µm are referred to as TiC45 and TiC23, respectively. The homogeneity of the distribution of the TiC in the powder blends is visually assessed from several micrographs. One representative micrograph can be found in the Appendix A, Figure A1. The flowability of the raw powders and powder blends is characterized using a GranuDrum™ from GranuTools (Awans, Belgium). The powders’ flowability is tested over a wide range of rotation speeds, and the flowability data is used to select an initial recoating speed for the LPBF process. Additionally, the powder layer absorptivity is measured for pure powders and powder blends, using a Spectrometer from Perkin Elmer (Lambda 950PE, Waltham, MA, USA) equipped with an integration sphere.

### 2.2. Laser Powder Bed Fusion Processing and Heat Treatment

**Figure A2:**
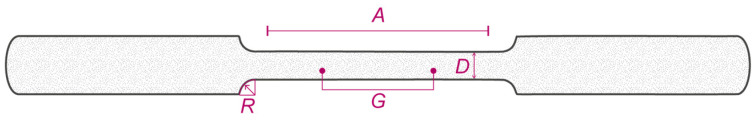
Schematic definition of the tensile samples’ geometry according to standard E8 [16]. The nomenclature of the different dimensions is defined in Table A2.

The LPBF process is performed using a commercial machine (TruPrint 1000) from Trumpf GmbH (Ditzingen, Germany), which is equipped with a 200 W fibre laser with a 30 µm laser spot size. The process is completed under argon atmosphere with an oxygen concentration limit of 100 ppm. Tensile specimens are prepared on a Ti64 substrate with the optimized laser parameters presented in Table 1 with the Volume Energy Density: VED=P/Vht, where laser power is P [W], the scanning speed is V [mm/s], the hatching distance is h [mm], and the layer thickness is t [mm]. The parameter optimisation is conducted in two consecutive steps: (i) the process window leading to densities above 99% is identified by varying laser powder, scanning speed and hatching distance; (ii) the laser powder and scanning speed are optimised (within the process window defined in (i)) to maximise the resulting as-built Young’s modulus and fracture strain. In addition, a second set of laser parameters is used for the sample’s contour to minimise surface roughness. This second set of parameters is less energetic than the one used for hatching in the inner region (referred to as “hatching region” from this point). A lower energy is used for the sample’s contour than for hatching in the inner region (referred to as the “hatching region” from this point) to reduce surface roughness. The laser scanning strategy is bi-directional with 67° rotation from one layer to another. The sample geometry follows the ASTM E8/E8M standard [16] (cylindrical specimen type 5, details are available in the Appendix A, Figure A2 and Table A2). The specimen surface is not post-treated to account for potential applications containing inaccessible regions due to part complexity. The samples are printed with their axis parallel to the building direction and in batches of 24.

Samples are then encapsulated in a quartz tube under an Argon atmosphere to avoid oxidation and nitridation and heat treated at 880 °C in a furnace from Nabertherm (Lilienthal, Germany). This temperature is selected to avoid crossing the β-transus while maximising C solubility and mobility. The heating and cooling rates are set at 600 °C/h and all samples are treated simultaneously. Four samples from each set are heat-treated for 24 h to ensure complete globularisation of TiC dendrites.

### 2.3. Microstructural and Mechanical Characterization

Samples are cut both along and across the building direction, then ground using SiC paper, and subsequently polished using several grades of diamond paste, and lastly, finished by OP-S solution (Struers, Copenhagen, Denmark). Low-magnification and high-magnification microstructural analysis is done by Scanning Electron Microscopy (SEM, FEI Scios 2 and ZEISS Merlin, Oberkochen, Germany) using Secondary (SE) and Backscattered Electron (BSE) imaging, as well as Electron Backscatter Diffraction (EBSD, Oxford Instruments, Abingdon, UK). General SEM uses an acceleration voltage of 10 kV, while EBSD uses a 25 kV acceleration voltage. The porosity of the sample is determined first by analysing low-magnification SE micrographs and, second, by X-ray Computed Tomography (XCT, Phoenix V|tome|x M from Weigate Technologies, Huerth, Germany). Image analysis considers a minimum of five micrographs per sample and uses an in-house developed Python script. The TiC reinforcement content is determined using the same image analysis method, from low-magnification micrographs when un-dissolved, and high-magnification micrographs when dendritic and granular. Etching provided by the OP-S solution suffices to clearly distinguish the Ti grains. Phase composition is characterized by X-Ray Diffraction (XRD, PANalytical X’Pert PRO (Tokyo, Japan) with PIXcel source at 45 kV and 40 mA). The variation of the C content in heat-treated TiC grains is evaluated by Scanning Transmission Electron Microscopy (STEM, Titan Themis, Thermo Fischer, Waltham, MA, USA) and Energy Dispersive X-Ray Spectroscopy (EDS) with an acceleration voltage of 200 kV. TEM lamellas are prepared in an SEM by a Focused Ion Beam (FIB, FEI Scios 2, Thermo Fischer, Waltham, MA, USA).

Tensile properties are characterized by as-built and heat-treated tensile samples at room temperature employing a 100 kN tensile machine (Zwick Serie E, Ulm, Germany) according to the ASTM 8M/E8M standard, using a strain rate of $8.33\times 10^{-5}\;s^{-1}$ [16]. The measurement of the strain during the test is performed by three means: with a Digital Image Correlation (DIC) system from GOM focused on one side of the sample, with a strain gage glued on the other side of the sample and with a 10 mm clip-on extensometer. On the one hand, the measurement of strain by multiple sensors allows one to compensate for a potential misalignment of the uniaxial system, and, on the other hand, to get complementary information about the localisation of the strain. Indeed, the strain gage measures the local strain of the region on which it is glued with a high sensitivity, while the extensometer measures the average strain over 10 mm, and the DIC enables the detection of potential strain localisation, in particular necking, over the whole reduced section. The machine strain rate is controlled using the clip-on extensometer. The tensile test is interrupted at 300 MPa and 700 MPa and unloaded to 100 MPa, producing two unload/reload cycles. This allows for measurement of the Young’s modulus and the overall elastic response of the sample. Data from at least 4 samples are averaged for each analysis. Finally, the fracture surfaces and cross-sections beneath them are observed by SEM to characterize failure mechanisms.

## 3. Results and Discussion

### 3.1. As-Built MMCs Microstructure

**Figure 3:**
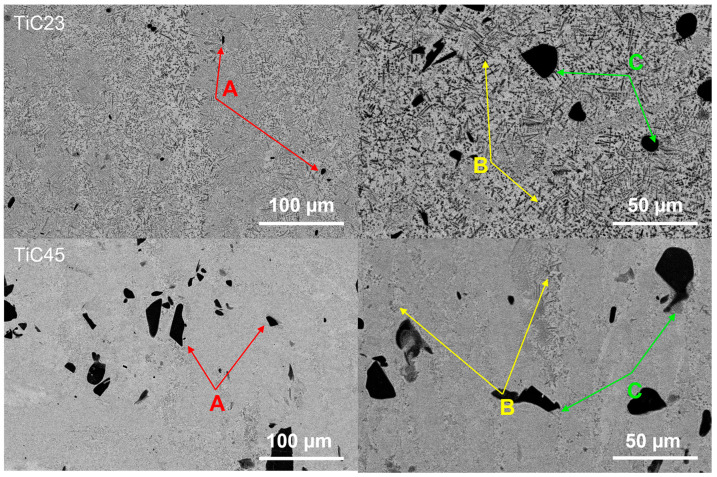
SEM micrographs presenting as-built microstructures for TiC23 (top) and TiC45 (bottom). Examples of un-dissolved TiC particles are indicated by the red arrows (A), primary dendritic TiC by yellow arrows (B) and secondary dendritic TiC by green arrows (C).

The microstructures of TiC23 and TiC45 in as-built conditions are studied by SEM. Figure 3 shows typical BSE micrographs of TiC23 (top) and TiC45 (bottom). It displays a light grey Ti-matrix hosting a homogeneous distribution of dark grey TiC dendrites (see arrows: yellow B and green C) as well as black un-dissolved TiC particles (red arrow A). Similar microstructures were observed by Gu et al. for similar TiC content in the powder mix [9]. Dendritic TiC is present in two subsets: (i) primary TiC precipitating from the liquid (yellow arrow B) and (ii) secondary TiC originating from the diffusion layer around partially dissolved TiC particles (green C). The temperature achieved during LPBF allows C from TiC to diffuse and enrich Ti liquid but melt pool liquid time. However, the presence of the remaining undissolved TiC indicates that the melt pool lifetime is not sufficient enough to dissolve the larger fraction of the TiC particles. This is reflected by the average particle size decreasing from 9.6 µm and 37.2 µm to 6.5 µm and 9.56 µm, for TiC23 and TiC45, respectively. The undissolved TiC particles account for 0.8 ± 0.5 vol% in TiC23 and 4.5 ± 0.4 vol% in TiC45. Similar effects were observed by Wang et al. in the LMD process, in which remaining particles sizes varied with the process parameters and the original powder size distribution [17].

**Figure 4:**
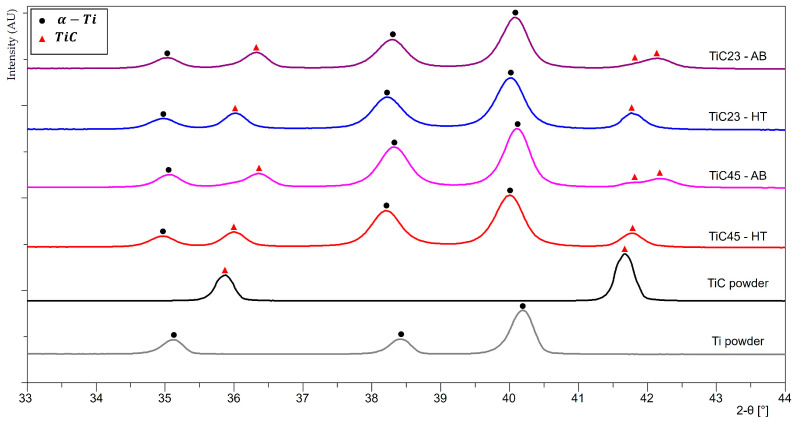
XRD patterns of powders feedstock, as-built LPBF TiC23 and TiC45, and heat-treated LPBF TiC23 and TiC45 with 2-θ angles between 33° and 44°.

**Figure A3:**
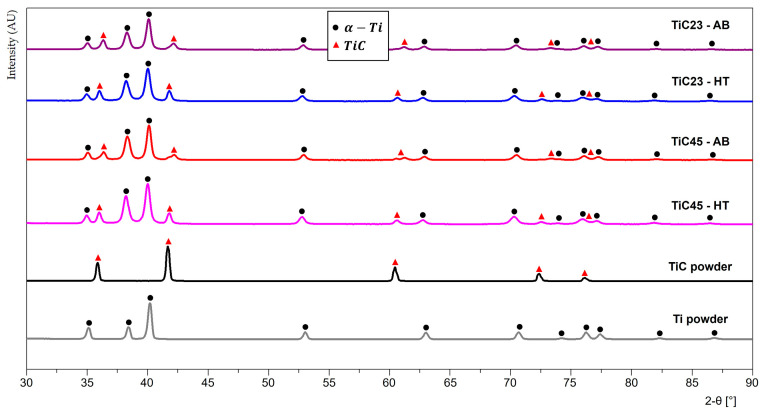
XRD patterns of powders feedstock, as-built LPBF TiC23 and TiC45 and heat-treated LPBF TiC23 and TiC45 with 2-θ angles from 58° to 76°.

**Figure A4:**
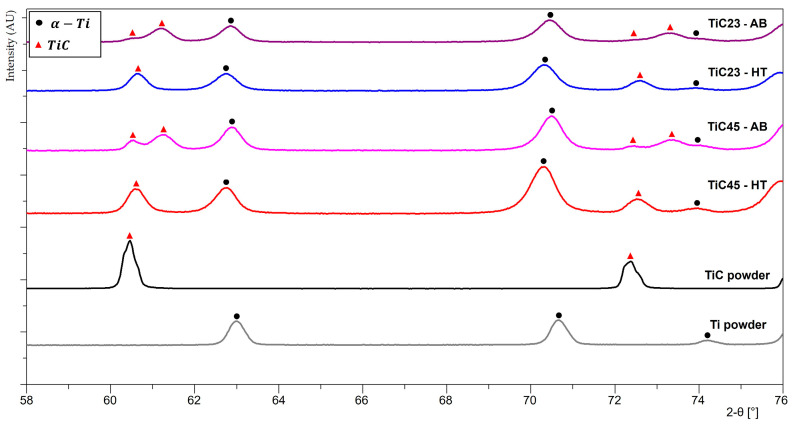
XRD patterns of powders feedstock, as-built LPBF TiC23 and TiC45 and heat-treated LPBF TiC23 and TiC45.

XRD patterns of the as-built LPBF MMC samples and feedstock powders, with selected 2-θ angles between 33° and 44°, are shown in Figure 4. Full XRD patterns as well as the 58–76° 2-θ angles regions are available in the Appendix A, Figure A3 and Figure A4. The Ti powder shows a typical hexagonal α-Ti pattern. However, α-Ti and martensitic α′-Ti are rather difficult to differentiate due to close unit cells and overlapping reflections of α and α′ phases. The rapid cooling induced in plasma atomization would most likely lead to martensitic α’-Ti. It is also reported by Haase et al. that pure Ti produced by Laser Powder Bed Fusion has a martensitic α’-Ti microstructure [18]. Both as-built LPBF TiC23 MMC and TiC45 MMC samples display α-Ti peaks as well as TiC peaks. The latter are shifted to higher 2θ angles compared to the raw stoichiometric TiC powder (e.g., the 35.91° peak is shifted to 36.34° for TiC23 and 36.38° for TiC45). It can be explained by variation in residual stresses, by a modification of the TiC lattice constant or a combination of the two phenomena. The TiC lattice constant $a_{TiC_{x}}$ is known to vary with the C/Ti ratio of TiC and was modelled empirically by Kurlov and Gusev, who fitted a second order polynomial on a large collection of literature data and obtained expression (1) [15].

(1) $$a_{TiC_{x}}=0.42055+0.02665x-0.01456x^{2}\pm 0.00005\;nm,$$

It is noted that the use of expression (1) is limited to cubic TiC_x_ and room temperature. In addition, TiC peaks at 42°, 61° and 73° are split into two peaks. Rietveld refinement is used to measure the average lattice constants of α-Ti and two TiC_x_ phases, as presented in Table 2. The lattice constant of the lower angle TiC peak subset is consistent with stoichiometric TiC particles, while the lattice constant of the TiC peak subset at a higher angle shift fits sub-stoichiometric TiC dendrites.

### 3.2. Heat-Treated Microstructure

**Figure 5:**
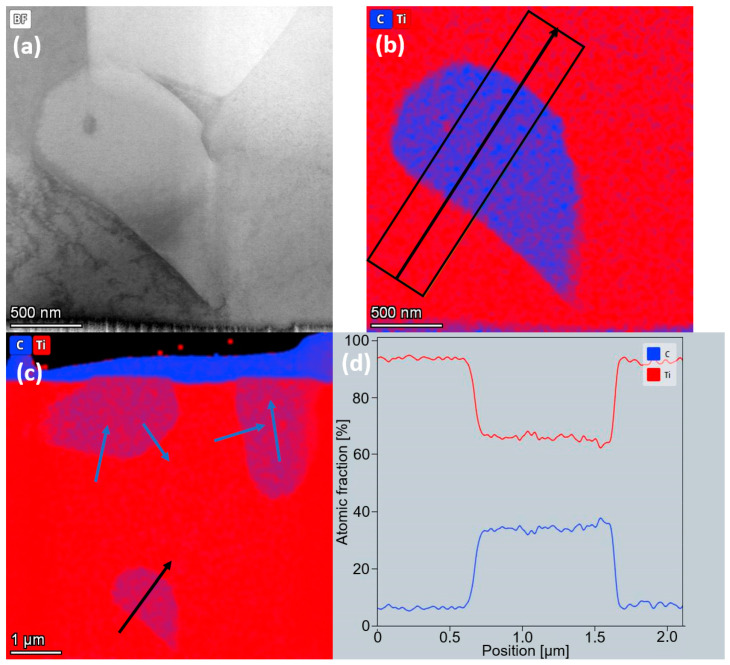
TEM-EDS Ti and C mapping of a TiC grain of sample TiC23 after 24h heat treatment (**a**) Bright-field micrograph of a TiC grain surrounded by Ti grains. (**b**) C (blue) and Ti (red) EDS mapping for the same region. The black arrow depicts the profile analysed and the average width used in the profile presented in (**d**). (**c**) C (blue) and Ti (red) overlay of the TEM lamella with arrows depicting the C and Ti profiles studied. Blue arrows refer to profiles not presented here.

Heat-treated microstructures of both TiC23 and TiC45 show a full conversion of the dendritic TiC into equiaxed TiC, while un-dissolved TiC particles do not present a significant geometrical evolution. The conversion of the TiC microstructure from dendritic to equiaxed is the result of the activation of several mechanisms thanks to the increased carbon solubility and mobility brought by the heat treatment. It can be explained by the dissolution and subsequent recrystallisation of a fraction of the dendritic TiC, as well as the outer layer of the un-dissolved TiC particles, by grain boundary migration thanks to higher atomic mobility, Ostwald ripening, or by a combination of the three. BSE contrast between un-dissolved TiC and equiaxed TiC is softer on average than the contrast between un-dissolved TiC and dendritic TiC, which seems to indicate a partial homogenization of the C/Ti ratio. The diffusion process appears to be far from being completed, as there is BSE contrast within the large un-dissolved TiC particles themselves. Further study of TiC grains by STEM-EDS displays a stable C content in grains resulting from the globularisation of dendrites, as presented in Figure 5. Conversely, in un-dissolved TiC grains, a decrease from the core to the edge of the TiC grain is observed. While semi-quantitative, these results indicate that the heat treatment homogenizes the C/Ti content in globularised TiC dendrites but is not sufficient to have the same effect on the un-dissolved TiC particles. The volumetric fraction of undissolved TiC grain decreases by 1.2 vol% in TiC45 and to a non-significant value in TiC23. Indeed, the heat treatment leads the smaller fraction of un-dissolved TiC particles to metallurgically bond to the matrix, as well as decreasing their stoichiometry by diffusion. This results in TiC equiaxed grains and previously un-dissolved TiC particles having the same size and contrast, and both can be considered as equiaxed TiC.

XRD analysis after heat treatment (Figure 4) does not show significant changes in the α-Ti phase. A small uniform shift of about 0.1° is measured for all Ti peaks, which is explained by the residual stress relaxation during the heat treatment. The increase in α-Ti lattice constants after heat treatment is characterized by a constant $c_{\alpha Ti}/a_{\alpha Ti}$ ratio, which is consistent with a relaxation of residual stresses and a constant C content at the solubility limit (0.08 wt% at room temperature in α-Ti). Indeed, variation in the content of C in solid-solution is known to affect the $c_{\alpha Ti}/a_{\alpha Ti}$ ratio [19]. All TiC peaks shift to lower 2-θ angles, and split peaks disappear. The transition from split TiC peaks in as-built conditions to single TiC peaks in heat-treated conditions indicates a shift from bimodal to monomodal distribution of the C content in TiC grains, i.e., heat treatment led to TiC’s C/Ti ratio homogenization at the sample level. The residual local variations of the C content in TiC grains observed by TEM are not resolved by XRD; thus, the TiC peak shift is explained by a lattice change induced by the redistribution of C atoms over the TiC phases and a relaxation of the thermal residual stresses. The comparison of the experimental TiC_x_ lattice constant with the one calculated from the C/Ti ratio using Kurlov and Gusev’s relation displays a reduction in the discrepancy between experimental and calculated lattice constants after the heat treatment, which is coherent with a reduction in the residual stresses of TiC [15]. Due to the empirical nature of Equation (1) and the single reflection used, it does not provide indications of the tensile or compressive nature or amplitude of said residual stresses.

**Figure 6:**
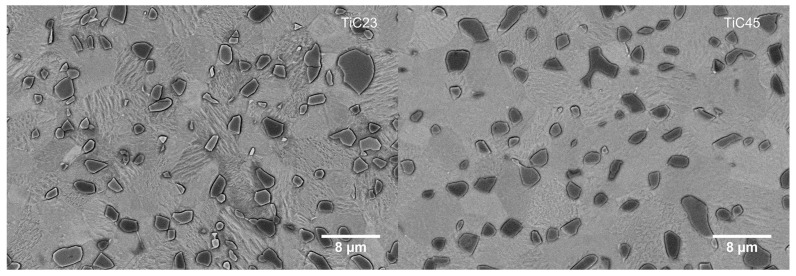
High-magnification BSE micrograph of typical microstructure found in TiC23 (**left**) and TiC45 (**right**) heat-treated samples. The grain of the α-Ti phase is visible in several light grey shades thanks to the grain orientation sensitivity of BSE and etching.

**Figure 7:**
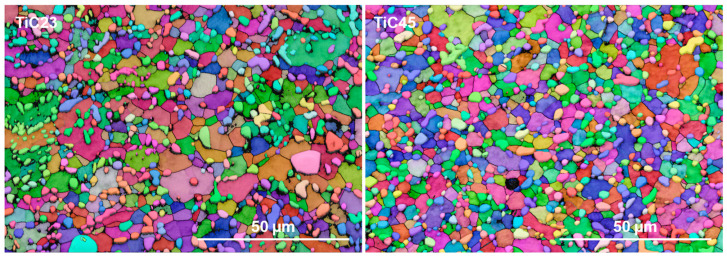
Typical EBSD maps of heat-treated TiC23 (**left**) and TiC45 (**right**). The different colours correspond to the crystalline orientation of the grain.

High-magnification micrographs of the microstructures, seen in Figure 6 indicate an almost full conversion of α’-Ti into α-Ti, with only a few remaining visible martensitic grains. EBSD maps at similar magnification (Figure 7) were used to statistically quantify Ti and TiC grain sizes. These analyses show that there is not a significant difference in grain size distributions between TiC23 and TiC45 (Ti grain size: 2.9 ± 1.6 µm and 3.0 ± 2.3 µm for TiC23 and TiC45; TiC grain size: 1.4 ± 0.9 µm and 1.2 ± 0.9 µm for TiC23 and TiC45). The minimal influence of the variation in feedstock properties over the microstructure dimension demonstrates tight microstructure control through the current LPBF process conditions.

Generally, heat-treated microstructures have enough contrast in BSE for image analysis measurement of reinforcement content using low-magnification micrographs. The effective reinforcement contents measured in TiC23 and TiC45 are 20.8 ± 5.5 vol% and 21.5 ± 4.7 vol%, respectively, which represents almost twice the nominal value (i.e., 12 vol%). This phenomenon is explained by the reduction in the C/Ti ratio in the carbides according to reaction (2).

(2) $$xTiC+Ti\overset{yields}{\to }(1+x)TiC_{x},$$

**Figure 8:**
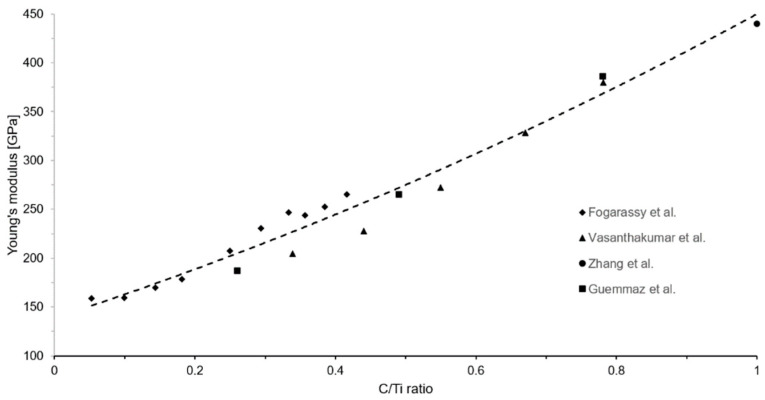
Young’s modulus of TiC_x_ as a function of the C/Ti ratio. Data were fitted with a second order polynomial function resulting in Equation (3) [20,21,22,23].

With *x* being the C/Ti ratio. Roger et al. showed stable C/Ti ratio of 0.57 after a 450 h heat treatment at 920 °C in a particulate Ti-TiC MMC with similar reinforcement content [13]. The elastic modulus displays significant variations as the C/Ti ratio evolve [20,21,22]. The method used by Kurlov and Gusev for shear modulus is reproduced by the authors to fit Young’s modulus experimental data from the literature with a second polynomial, as presented in Figure 8 [15]. This led to the empirical Equation (3), which estimates the variation of TiC_x_’s Young’s modulus $E_{TiC_{x}}$ at room temperature as a function of the C/Ti ratio x.

(3) $$E_{TiC_{x}}=138.74+234.04x+77.21x^{2}\;\pm \;16\;GPa,$$

Using this relation and the C/Ti ratio calculated from XRD measurements, the average Young’s moduli for the TiC_x_ phases in heat-treated TiC23 and TiC45 are estimated to be 361 ± 16 GPa and 379 ± 16 GPa, respectively. The impact of the variation of the Young’s modulus and reinforcement content with the C/Ti ratio is discussed in a dedicated section with the experimental mechanical properties.

### 3.3. Density Characterization

**Figure A5:**
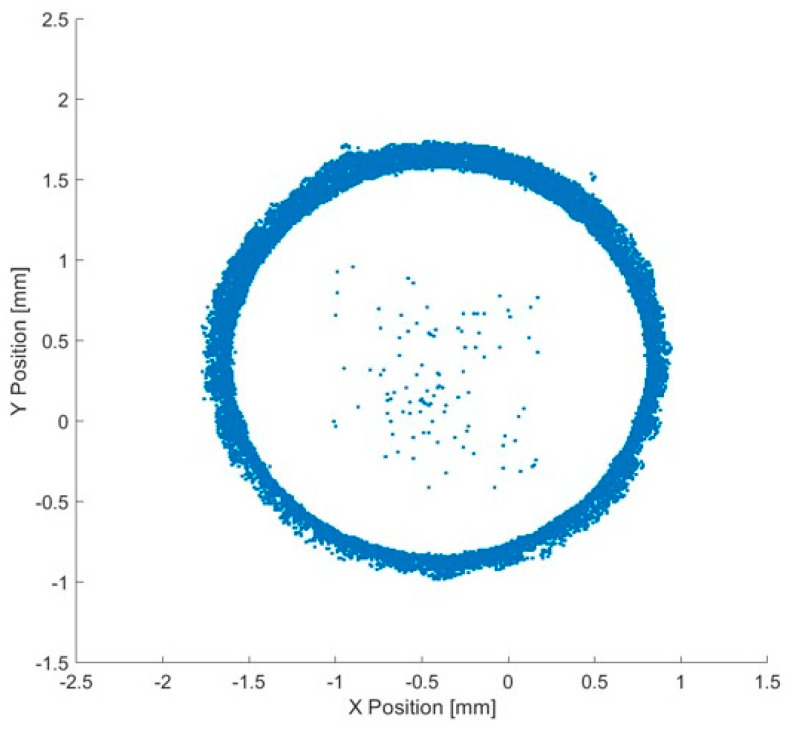
Integrated porosity positions over the whole reduced region of one TiC23 tensile sample.

The density is analysed globally in the sample, as well as in the hatching region and contour. The porosity analysis done using image analysis of micrographs shows virtually no porosity in the hatching region, but shows an increased number of defects close to the edges of the samples. XCT allows a better understanding of the spatial distribution of porosity and displayed that a majority of the porosities reside at the interface between the hatching region and the contour (Appendix A, Figure A5); hence, the “hatching density” is measured by removing all porosity outside of the hatching regions. Hatching and sample densities are compared in Table 3, indicating that the porosities outside the hatching region account for a loss of 0.3 to 0.4% density in TiC23 and TiC45, respectively; thus, further parameter optimization of this region could lead to a density close to 99.9% for TiC23. On the other hand, for TiC45, most of the porosity is located at other locations. One needs to note that the XCT measurements conducted have a resolution of 20 µm, which restricts this conclusion to porosities larger than this size.

### 3.4. Mechanical Properties

**Figure 9:**
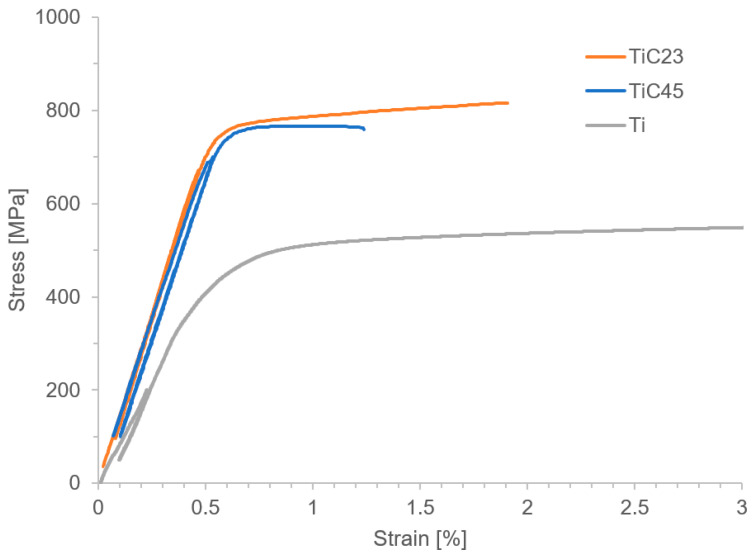
Stress–strain curves of typical heat-treated TiC23 and TiC45 as well as as-built Ti samples.

Mechanical characterization, summarized in Table 4, shows a strong increase in Young’s modulus and Ultimate Tensile Strength (UTS) in all MMCs compared to pure Ti. While as-built MMCs are brittle and do not yield, both heat-treated TiC23 and TiC45 deform plastically, enough to enable the measurement of the 0.2% Yield Strength (YS), as demonstrated in Figure 9. The fracture strain is twice as large after the heat treatment and reaches a similar value to that reported by Yan et al. in the case of TiC45 and is 30% above it in the case of TiC23. The lower fracture strain presented by TiC45 is assumed to be due to the increase in stochastic defect density with particle size observed in ceramics. This demonstrates the effectiveness of the heat treatment used in improving the ductility of Ti-TiC MMCs. It is assumed that both the reduction in the local stress concentration (owing to the conversion of dendritic TiC to granular TiC) and the normalization of the stress field (through the homogenization of the TiC particles’ stoichiometry and size) lead to the ductility improvement observed. The UTS is also observed to increase after the heat treatment, while strength is generally known to decrease with an increase in the average grain size. In this case, the transition from a brittle material to a relatively ductile material enables the increase in UTS. Indeed, brittle materials such as ceramic often fail below their theoretical strength due to high sensitivity to defects. The presence of intrinsic defects in undissolved particles in the as-built MMCs leads to critical failure before reaching composite theoretical strength. On the other hand, in heat-treated conditions, matrix plasticity and strain hardening enable deformation even in the presence of cracked particles. It results that the peak strength of this MMC is reached at the brittle-ductile transition and that longer heat treatments most likely lead to a decrease in strength. In heat-treated MMCs, the YS was increased by 37% and 57% for TiC23 and TiC45, respectively, and the UTS by 31% and 44%, respectively. As a comparison, Yan et al. achieved a 60% and 32% increase in YS and UTS, respectively, with the addition of 0.5 wt% graphene in Ti-6Al-4V produced by LPBF and a 12% and 9% increase, respectively, in the same MMC produced by SPS [11]. Lagos et al. [3] achieved a 20% YS increase and 14% UTS increase in Ti-6Al-4V reinforced by 10 vol% TiC. While the strengthening observed in this study is at the same level as the one achieved by Yan et al., the difference in reinforcement content needed to achieve these values demonstrates the difference between MMCs focusing on strengthening and stiffness improvement. Indeed, five strengthening mechanisms describe strength increase: Hall–Petch, Orowan, thermal coefficient mismatch, carbon solid-solution and load transfer. These well-known mechanisms and their distribution will not be detailed here, as strength is not the focus of this study. However, one can point out that: (i) Hall–Petch, Orowan and CTE mismatch strengthening mechanisms are inversely proportional to reinforcement size favouring smaller reinforcements; (ii) load transfer is dependent on the aspect ratio of the reinforcement particles; and that (iii) CTE mismatch is relatively low in Ti-TiC [11]. Stress–strain curves in Figure 9 show a stronger strain hardening for TiC23 compared to TiC45. More experiments would be needed to define its precise origins; however, a few elements can be proposed. Larger un-dissolved particles could lead to more strain localization, as the stress plateau of TiC45 may indicate, and therefore earlier failure. On the other hand, smaller particles in TiC23 would increase their number and enhance dislocation–particle interactions (Orowan mechanism).

The Young’s modulus does not appear to be affected by the heat treatment with all values slightly below 150 GPa. This elastic modulus is known to be influenced mainly by the properties of the components of the composites and their relative fraction, as seen in the parameters used in standard laws of mixture such as Hashin–Shtrickman bounds [24]. In addition, in the case of elongated reinforcements, the orientation of the reinforcement can also play a significant role. However, considering the low aspect ratio of the TiC_x_ grains observed in heat-treated conditions, this factor can be neglected in this study. The absence of significant variation in the Young’s modulus during the heat treatment indicates that, on average, the variations in lattice constants, C/Ti ratios and, hence, elastic moduli are compensated by the variation of reinforcement contents. This concept can be visualized by using Hashin–Shtrickman bounds for Ti-TiC with C/Ti ratios of 1 and 0.8. The TiC Young’s moduli were calculated using the empirical relation proposed in this study (3) and the Poisson ratios $\nu_{TiC_{x}}$ using the empirical relation (Equation (4)) proposed by Kurlov and Gusev [15].

(4) $$\nu_{TiC_{x}}=0.256-0.065x\;\pm 0.002$$

**Figure 10:**
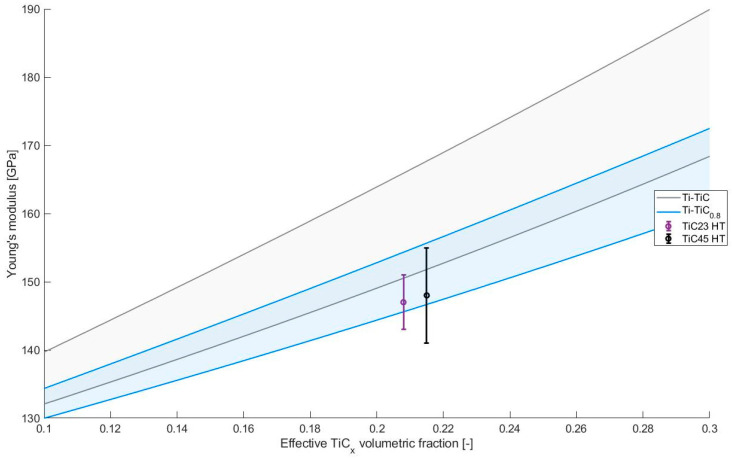
Young’s modulus estimated by Hashin–Shtrickman bounds for Ti-TiC as a function of the TiC volumetric content [24]. Grey, green and blue regions provide the estimates for different C/Ti ratio in TiC. Experimental data from TiC23 and TiC45 in heat-treated conditions are visible in purple and black, respectively.

The resulting Hashin–Shtrickman bounds are presented in Figure 10 along with the Young’s modulus measured for TiC23 and TiC45 in heat-treated conditions. The reduction in the C/Ti ratio leads to a decrease in the expected properties. It is clearly visible in Figure 10, with the blue (Ti-TiC_0.8_) regions being lower than the grey one (Ti-TiC). The experimental values for TiC23 and TiC45 fit on the bottom bound of their relative C/Ti ratio, which is coherent with the current microstructure as the lower bound is defined as a homogeneous matrix reinforced by a homogeneous distribution of spherical inclusions of reinforcement material. The definition of the higher bound is instead opposite by definition, implying that the reinforcement material contains a homogeneous distribution of spherical inclusions of matrix materials; the latter is conceptually closer to cermets than MMCs. It is important to note that these considerations were made considering effective reinforcement content. If only nominal reinforcement was considered, experimental data would be above the higher bound of stoichiometric Ti-TiC (12 vol% and 147 GPa and 148 GPa). As introduced with expression (2), the conversion of stoichiometric TiC into TiC_x_ leads to an increase in the reinforcement content, which cannot be neglected, e.g., in this case, where effective reinforcement content is almost twice the nominal reinforcement content.

The Young’s moduli measured with the unload at 300 MPa (E300) and 700 MPa (E700) do no present significant variations, which indicates the likely absence of damage or pre-existing defects in the composites. Considering the Young’s moduli at the end of the process, TiC23 and TiC45 present a modulus enhancement of 26% compared to unreinforced Ti.

**Figure 11:**
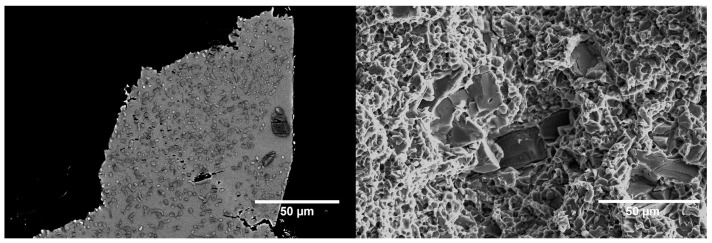
Fracture cross-section (**left**) and fracture surface (**right**) micrographs of a TiC23 sample after tensile failure. The fracture cross-section was extracted at the edge of the sample.

Fracture surface analysis, illustrated in Figure 11, shows a ductile matrix with distinctive ductile dimples together with a distribution of brittle TiC un-dissolved particles. TiC_x_ grains originating from the precipitated TiC dendrites were identifiable on the cross-sections perpendicular to the fracture surfaces. A study of both fracture surfaces and sub-surface cross-sections indicates the presence of two failure mechanisms: (i) particle cracking and (ii) particle delamination. A crack can be seen progressing from one TiC_x_ grain to another through a deformed matrix, as well as cracked TiC_x_ grains and TiC un-dissolved particles close to failure points. The analysis of the full fracture surface does not show a propensity of the failure path to follow the cracked un-dissolved TiC particles. Indeed, almost none of them are found on the fracture surface of TiC23, and very few on the fracture surface of TiC45; however, the exact failure mechanism as well as the mechanism controlling the onset of failure are not fully understood at this point.

## 4. Conclusions

Ti-TiC MMCs with a 12 vol% reinforcement content are successfully produced using LPBF and subsequent heat treatment. Two grades of TiC powder size distribution are used, and the microstructure and mechanical properties are thoroughly characterised and investigated.

- A density > 99% is achieved with the lowest reinforcement particle sizes, while a density >98% is achieved with the other one.
- A drastic evolution of the reinforcement content (12 vol% to 21 vol%) and C/Ti ratio (0.98 to 0.8) is observed following LPBF and heat treatment. The effect of the variation of TiC elastic properties and volumetric fraction on composite elastic properties is discussed.
- Young’s moduli of both MMCs are 26% higher (149 GPa) than the one of Ti grade 2.
- The fracture strain after heat treatment is more than twice the one observed in as-built conditions thanks to the conversion of dendritic TiC_x_ into equiaxed TiC_x_.
- The elastic modulus is not affected by the heat treatment. It is proposed that the decrease in TiC’s elastic modulus induced by the reduction in the C/Ti ratio is compensated by the increase in the TiC_x_ volumetric fraction.
- Lower particle sizes are shown to be beneficial for both density and mechanical properties and are correlated to a lower fraction of un-dissolved TiC particles. In addition, ductility increase after heat treatment is more pronounced for the latter.
- Fracture up to 1.7% is achieved after heat treatment and for lower particle size distribution. The value is 30% higher than previously reported for Ti-based MMCs produced by LPBF with a similar elastic modulus. This improvement demonstrates the potential of heat-treated Ti-TiC and is a first step in enabling its use in stiffness-driven applications.

## Figures and Tables

**Table 1:** Laser parameters used in LPBF processing. Contour distance refers to the minimum distance between a laser pass from the contour and the hatching region. Contour number accounts for the thickness of the contour in terms of the number of laser passes.

Hatching Region		Contour	General Parameters		
VED [J/mm^3^]	Hatching distance [µm]	VED [J/mm^3^]	Layer thickness [µm]	Contour distance [µm]	Contour number [-]
166.7	40	52	30	30	1

**Table 2:** Crystallographic parameters of TiC45 and TiC23 in as-built and heat-treated conditions. * Calculated from C occupancy in TiC_x_ [15].

Parameters	TiC45 AB	TiC23 AB	TiC45 HT	TiC23 HT
$a_{\alpha Ti}$ [Å]	2.957	2.956	2.961	2.961
$c_{\alpha Ti}$ [Å]	4.698	4.698	4.707	4.706
$c_{\alpha Ti}/a_{\alpha Ti}$ [-]	1.589	1.589	1.590	1.589
$a_{TiC_{x},\;exp}$ [Å]	4.279	4.282	4.319	4.318
$a_{TiC_{x},\;calc}$ * [Å]	4.295	4.308	4.324	4.326
$C_{TiC_{x}}\;occupancy$ [-]	0.45	0.55	0.76	0.81
$a_{TiC,\;exp}$ [Å]	4.321	4.310	x	x
$a_{TiC,\;calc}$ * [Å]	4.326	4.326	x	x
$C_{TiC}\;occupancy$ [-]	1.00	1.00	x	x

**Table 3:** XCT density measurements. Sample density refers to the overall density of the sample. Hatching density is defined as the density of the inner region of the sample. It is calculated by removing the skin zone of the sample during the analysis.

Sample	Sample Density [%]	Hatching Density [%]
TiC23	99.5	99.9
TiC45	98.8	99.1

**Table 4:** Tensile properties of TiC23 and TiC45 MMC samples compared to those of the Ti reference.

	TiC45 AB	TiC45 HT	TiC23 AB	TiC23 HT	Ti
E300 [GPa]	153 ± 4	148 ± 7	149 ± 17	147 ± 4	117 ± 7
E700 [GPa]	-	149 ± 9	-	149 ± 3	-
YS [MPa]	-	700 ± 74	-	806 ± 20	512 ± 6
UTS [MPa]	668 ± 21	752 ± 11	778 ± 15	827 ± 9	576 ± 4
e [%]	0.5 ± 0.0	1.3 ± 0.1	0.7 ± 0.1	1.7 ± 0.2	29.3 ± 3.1

## Data Availability

Data will be made available on request.

## Appendix A

**Table A1:** Ti and TiC chemical composition.

	Ti [wt%]	C [wt%]	O [wt%]	H [wt%]	N [wt%]	Fe [wt%]	Other [wt%]
Ti grade 2	balance	0.01	0.14	0.002	0.01	0.02	<0.2
TiC	balance	19.6	0.5	-	-	0.2	<0.01

**Table A2:** Dimension of the tensile sample according to ASTM E8 as well as the dimension of the printed samples [16].

	ASTM E8 [mm]	Effective Dimensions [mm]
G—Gauge length	10 ± 0.1	10
D—Diameter	2.5±0.1	2.5
R—Radius of filler, min	2	2
A—Length of reduced parallel section, min	16	25
